# Differences of Opinion

**Published:** 1908-03

**Authors:** 


### Abstracts and Selections.

### Differences of Opinion.

Differences of opinion are bound to exist in medical affairs. There are so many ways of going forward, and only one of standing still. If the medical profession were standing still, there would be absolute agreement, for there could be nothing else. Here is our good friend Abbott exploiting with all his energy his H. M. C., and the *Journal of the A. M.* is hitting him right and left concerning it. The *Journal* claims that it is not a discovery; that it is a nostrum, and inert. Added to this contumely, it keeps its columns closed to Dr. Abbott's protests, and when it occasionally permits him to answer its most violent attacks, it draws the points from his articles, and in a top-loftical manner asserts that certain parts of his article were not necessary to publish. It has a masterful, domineering, tyrannical manner of acting, something like the powerful rulers of the Old World, who do about as they please. It does not add to its reputation, or that of its conductor, for fairness. Perhaps it does not care for those things. Perhaps the ability to tyrannize is what it is after; perhaps its conductor would rather live in the memory of his contemporaries as being a tyrant, unfair and oligarchic, than to have any other sort of a reputation.

Some day the *Journal*, if the trustees of the future are wise, will have a different sort of a conductor to do the everyday work. He may not make more money for the A. M. A. than Dr. Simmons is capable of doing; but he will leave a sweeter name for himself, and those who have swords to cross with him will remember him with better feelings than they will ever remember Dr. Simmons. It may be that some individuals are engaged in the business of working the profession in an unprofessional, unethical manner. It may be that some preparations which are being advertised and offered the medical men of this country are worthy of severe criticism. But there is a high-handed method of treating manufacturers, who are believed by the rank and file of the profession to be engaged in making excellent preparations; and the feeling is that a coterie of persons, masquerading under some high-sounding name as a committee, are making fish of one set of manufacturers and fowl of others; and that all this is being done, more or less, under the manipulation of the men temporarily at the *A. M. A. Journal* helm.

Then there is the attack that Dr. McCormack makes, to the right and the left. He is taken to task by the National Association of Retail Druggists. Dr. McCormack has charged this organization with systematically opposing all efforts in Legislatures to secure the passage of pure food laws; with introducing decoy bills, and spending money, solely for the purpose of placing obstacles in the way of legislation beneficial for the public. It rather seems that the secretary of the N. A. R. D. has got Dr. McCormack in a corner, and he insists that if Dr. McCormack was accountable for his actions at the time he made his most objectionable statements, he was a knave or a fool.

It is a pity that discussions of such matters can not be conducted with calmer feelings. There is something unscientific in a method which calls names. And yet, however scientific one may be—however much one's life may be devoted to the art of discovery—there is a good deal of human nature left, and when one is unjustly charged with unworthy conduct, there is a strong disposition to strike back with the most forcible language at command. One would like to be able to believe that the criticisms made by the men who have temporary control of the A. M. A. were actuated by excellent motives, and that these men were paragons of wisdom. But, alas! this is not the general opinion. There is much that is strictly human in what these men do. They are true descendants of the old Adam. However much the system under which they are elected to their places may appear to be an ideal system, the cloven foot too frequently shows through; and if Dr. Simmons and Dr. McCormack are belabored strenuously from one end of the country to the other, let us not believe that they are martyrs, suffering for the good of a cause whose flag they uphold with a single eye to the welfare of the profession, and without a particle of regard for their own aggrandizement.—*Medical Sentinel.*

---

Uric Acid Diathesis.—Haig advises a vegetable diet with milk, and the avoidance of meats, especially young flesh and soups—also herrings, eggs, tea, coffee and cocoa. Moderate exercise is helpful. Iodide of mercury, followed by or alternated with salicylates, may be given for some time.—*Denver Med. Times.*

